# Extraction of Phenolic Compounds from Fresh Apple Pomace by Different Non-Conventional Techniques

**DOI:** 10.3390/molecules26144272

**Published:** 2021-07-14

**Authors:** Luna Pollini, Lina Cossignani, Cristina Juan, Jordi Mañes

**Affiliations:** 1 Department of Pharmaceutical Sciences, Food Science and Nutrition Section, University of Perugia, 06126 Perugia, Italy; luna.pollini@studenti.unipg.it (L.P.); lina.cossignani@unipg.it (L.C.); 2 Laboratory of Food Chemistry and Toxicology, Faculty of Pharmacy, University of Valencia, 46100 Burjassot, Spain; jordi.manes@uv.es

**Keywords:** apple pomace, polyphenols, phloridzin, non-conventional extractions, UAE, UTE, ASE, PEF, Q-TOF-LC/MS

## Abstract

Red Delicious apple pomace was produced at laboratory scale with a domestic blender and different non-conventional extraction techniques were performed to isolate phenolic compounds, such as ultrasound-assisted extraction (UAE), ultraturrax extraction (UTE), accelerated solvent extraction (ASE) and pulsed electric field (PEF) extraction pre-treatment. Total phenolic content (TPC) was determined by Folin–Ciocalteu assay. Phloridzin, the main phenolic compound in apples, was determined by chromatographic analysis Q-TOF-LC/MS. The results obtained with these techniques were compared in order to identify the most efficient method to recover polyphenols. The highest value of TPC (1062.92 ± 59.80 µg GAE/g fresh apple pomace) was obtained when UAE was performed with EtOH:H_2_O (50:50, *v*/*v*), while ASE with EtOH:H_2_O (30:70, *v*/*v*) at 40 °C and 50% of flush was the most efficient technique in the recovery of phloridzin. The concentration of the main phenolic compounds ranged from 385.84 to 650.56 µg/g fresh apple pomace. The obtained results confirm that apple pomace represents an interesti-ng by-product, due to the presence of phenolic compounds. In particular, phloridzin could be considered a biomarker to determine the quality of numerous apple products. Therefore, this research could be a good starting point to develop a value-added product such as a functional food or nutraceutical.

## 1. Introduction

In recent years, apple has been considered one of the most widely consumed fruits in the world, due to its widespread geographical and seasonal availability. Moreover, the consumers of apples cover a large age range, starting from infants, who eat them as apple purée or compote, to adolescents, as mainly apple juice, and to adults as fresh fruit.

The well-known health benefits of fruit against cardiovascular diseases are due to polyphenols, considered the main compounds responsible for their cardioprotective, anticancer and chemopreventing effects [1]. In fact, the main phenolic compounds in apple, phloridzin and chlorogenic acid, have antioxidant properties that contribute to the prevention of inflammation and hyperglycaemia and many diseases affecting vascular function, blood pressure and hyperlipidemia [2]. Moreover, the important role against diabetes of the phenolic compounds of the apple, for their anti-hyperglycemic effects, has also been reported [3]. These authors claimed the ability of young apple’s polyphenols to retard the postprandial blood glucose and insulin levels in mice for both acute and 1-week intervention trials. In fact, phloridzin is a well-known competitive inhibitor of glucose transporters (SGLT1 and SGLT2) through the binding of the glucose moiety to the Na+/glucose co-transporter [4].

The main phenolic compounds found in apple are hydroxycinnamic acids; flavan-3-ol monomers; flavan-3-ol polymers, also called procyanidins; dihydrochalcones; flavonols, and anthocyanidins. Among them, the most represented are chlorogenic acid as a hydroxycinnamic acid, catechin and epicatechin as flavan-3-ol monomers, and phloridzin as a dihydrochalcone [5]. It is also important to consider the different distributions of phenolics in different parts of the fruit, such as the flesh, seeds, leaves, and skin. For example, dihydrochalcones concentrate in the core and the seeds, while the skin is also rich in flavonols and anthocyanins. Moreover, differences in the cultivar, area of cultivation, maturity, storage, extraction procedures, analytical techniques, and pre- or post-harvest factors could affect the amounts of polyphenols found in apples [6].

Recently, the green economy and the recycling trends are having a great impact on industries, particularly food industries, where the amount of waste represents an important management problem. The main and most common challenge is to recover bioactive compounds, in order to reuse the food waste and develop functional foods using these value-added products, with beneficial effects on human health [7].

Apple pomace is one of the most abundant wastes; in fact, 10 million tons are produced every year worldwide. It consists of pulp, peels, seeds, and stalks generated from apple juice production [8]. The use of apple pomace for fortification purposes has been investigated in bakery products, such as cakes, muffins, cookies, bread, biscuits, crackers, and extruded snacks; in the enrichment of dairy products, such as yoghurt and ice cream; in apple juice, and in meat products such as chicken patties and beef jerky [9,10].

Regarding the recovery of phenolics from vegetable waste, one of the most important phases is the extraction technique. Nowadays, non-conventional extraction methods such as ultrasound-assisted extraction (UAE), microwave-assisted extraction (MAE), supercritical fluid extraction (SFE), pressurized liquid extraction, or accelerated solvent extraction (PLE or ASE), are being increasingly used, showing several advantages over conventional extraction methods such as maceration, decoction, or Soxhlet. In particular, the main advantages of non-conventional in respect of conventional techniques are the better yield and the time, cost, and solvent savings, due to the better penetration of the solvent into the vegetable matrices, while the main disadvantage is the possible degradation of phenolic compounds, due to the heat generation during the extraction [7]. However, considering the structural differences of the numerous categories of phenols present in a plant product, it is necessary to develop an extraction method that guarantees high extraction yields for the different phenols and, at the same time, is fast, economical, and environmentally friendly [11,12].

The aim of this research was to investigate the extraction of phenolic compounds from Red Delicious apple pomace, with different non-conventional extraction techniques such as UAE, UTE, ASE, and PEF, using EtOH:H_2_O (50:50, 70:30, and 30:70, *v*/*v*), in order to establish the most efficient and powerful extraction method. Therefore, the determination of total phenolic content was carried out using a colorimetric method based on Folin–Ciocalteu reagent. Afterwards, the analysis of phloridzin, one of the main phenolic compounds in apple, was performed by ultra-high-performance liquid chromatography quadrupole time of flight coupled to mass spectrometry (Q-TOF-LC/MS).

## 2. Results and Discussion

### 2.1. Total Phenolic Content of UAE, UTE, ASE, and PEF Extracts from Fresh Apple Pomace

**Figure 1:**
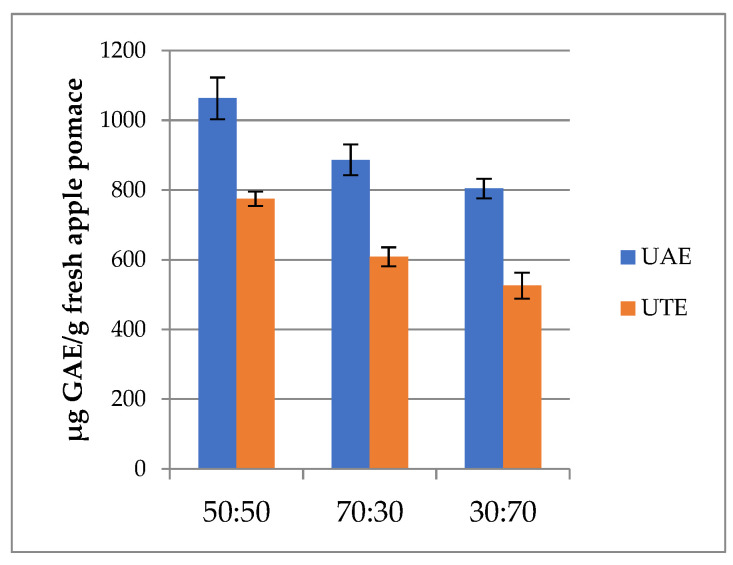
TPC results of UAE and UTE extracts. Error bars represent the SD.

**Figure 2:**
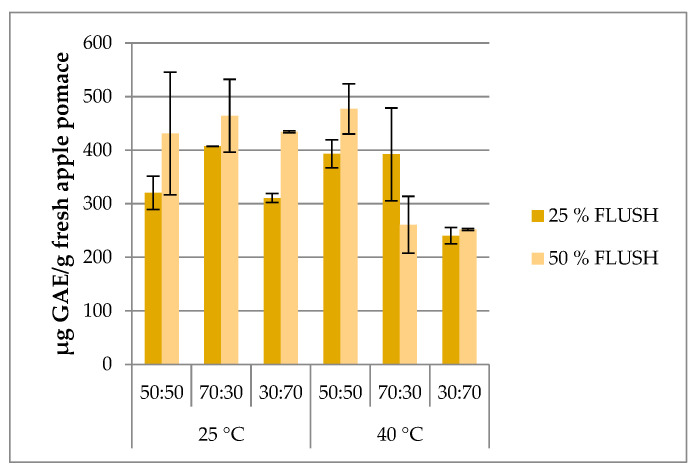
TPC results of ASE extracts. Error bars represent the SD.

**Figure 3:**
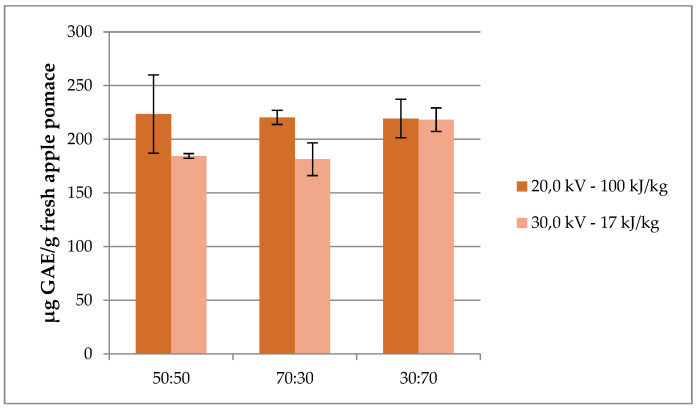
TPC results of PEF pre-treated extracts. Error bars represent the SD.

To investigate the efficiency of different non-conventional extraction techniques, fresh apple pomace extracts (FAPE) were subjected to analytical procedures for determining the phenolic compounds. Firstly, spectrophotometric determination with Folin–Ciocalteu reagent was performed to quantify the TPC through a redox reaction. Figure 1, Figure 2 and Figure 3 show the content of TPC, expressed as µg GAE/g of fresh apple pomace, for the different extraction techniques (UAE, UTE, ASE, and PEF). The results are reported in the graphs as mean ± standard deviation of two replicates. Considering the TPC values, UAE (Figure 1) gave the highest amount; in particular, the FAPE extracted with EtOH:H_2_O (50:50, *v*/*v*) showed 1062.9 µg GAE/g of fresh AP. Lower TPC content was observed for EtOH:H_2_O (70:30, *v*/*v*) extract and even lower when EtOH:H_2_O (30:70, *v*/*v*) was used as the extraction solvent. Nowadays, UAE is one of the most frequent techniques carried out for bioactive compound extraction, due to the fact that it uses common laboratory equipment. Moreover, the ability of ultrasound waves to disrupt the cell membrane of vegetal tissues is well-known, improving the recovery of bioactive compounds from plant foods and by-products [13].

The results obtained in this study were quite in accordance with the values (150–1200 mg GAE/100 g DM) reported by Wang et al. [14], who isolated phenolic compounds from the flesh and peel of Granny Smith and Red Delicious apples through ultrasound-assisted aqueous extraction, using a titanium ultrasonic probe at different specific energy inputs. Figure 1 shows also that FAPE obtained with ultraturrax had lower TPC content than UAE extracts, with values ranging from 525.7 to 774.6 µg GAE/g of fresh apple pomace and showing the same trend observed for UAE extract, regarding the ethanol:water ratio of the extraction mixture. Ultraturrax is a homogenizing, emulsifying, and suspending tool, but it is also used in the extraction of plant material. In the literature, a successful extraction method coupling UTE with UAE (UT–UAE) has been described, improving yield and decreasing extraction time [15,16]. Furthermore, UTE is suitable for the analysis of pesticides too, as reported by Sturm et al. [17].

Santarelli et al. [18] reported higher TPC when studying UTE extracts from the pulp of organic and *Malus domestica* cv. Golden Delicious apples, considering pre-treatment with dipping and vacuum impregnation in lemon juice solution, and different storage conditions, including freezing (3.8–6.3 mg GAE/g DM). However, it should be noted that their results were expressed as dry weight, while in the present work, we use the fresh matrix.

In this investigation, ASE was used as an alternative non-conventional technique to extract phenolic compounds from apple pomace. ASE is a non-conventional time-saving extraction technique, where pressure is one of the most important parameters. In fact, the high pressure allows us to increase the penetration power of the solvent into the matrix, increasing the time of contact between sample and solvent. ASE has been used to extract a wide variety of compounds and samples, such as herbal samples, dietary supplements, and nutraceuticals, saving vast amounts of time and solvent [19]. The elevated temperatures improve the extraction performance due to the increase in the mass transfer effect and the destruction of surface equilibrium [20].

The results obtained for ASE extracts from apple pomace, reported in Figure 2, showed quite homogeneous TPC values. Higher efficiency (from 310.7 to 464.2 µg GAE/g of fresh apple pomace) was obtained at 25 °C with 50% or 25% of flush and with all solvents and proportions proven; however, at 40 °C, for EtOH:H_2_O (70:30 and 30:70, *v*/*v*), the extracts presented less extractive effectiveness. The 50:50 hydroalcoholic mixture was confirmed to be the best extraction solvent, as visible for the extract obtained with EtOH:H_2_O (50:50, *v*/*v*) at 40 °C and 50% flush (476.8 µg GAE/g of fresh apple pomace), even if a similar TPC content was determined in the extract obtained with EtOH:H_2_O (70:30, *v*/*v*) at 25 °C with 50% of flush (464.2 µg GAE/g of fresh apple pomace). Franquin-Trinquier et al. [5] used an ASE experimental design using different solvents (methanol, acetone:water 70:30, or both) on freeze-dried powder of Braeburn apple pulp, and they reported values ranging from 2240.5 to 3348.5 mg catechin/g fresh fruit, amounts of TPC higher than our results.

Lastly, PEF was also tested in this study as a pre-treatment to extract phenolic compounds from apple pomace. PEF is a processing technology that consists in the application of short electric field pulses of high intensity to the matrix between the two electrodes. The induction of a transmembrane potential difference can result in the electroporation of the cell membrane, increasing the permeability of the cytoplasmatic membranes, which results in the easier release of the intracellular contents [21]. The main advantages of this treatment are the low energy consumption as well as the low increasing temperature. It has been proven that PEF treatment noticeably increases the amount of polyphenols extracted and their antioxidant activity [22]. TPC results obtained for PEF pre-treated samples (181.4–223.5 µg GAE/g of fresh apple pomace) are shown in Figure 3.

It can be observed that the values were very similar considering the two conditions set (20.0 kV–100 kJ/kg and 30.0 kV–17 kJ/kg), regardless of the hydroalcoholic mixture used for solvent extraction. These results are lower than those obtained by Lohani and Muthukumarappan [21], who carried out PEF pre-treatment on fermented apple pomace powder, to release the bound phenolics (402.7 mg GAE/100 g dry weight). However, it should be taken into account that the results of this study were reported on fresh weight.

Considering all the obtained results, it could be noticed the highest TPC value of FAPE was with ethanol:water at 50:50 (*v*/*v*), in all techniques carried out, except for PEF pre-treated (30.0 kV–17 kJ/kg), for which EtOH:H_2_O (30:70, *v*/*v*) was the solvent with the best recovery of TPC.

### 2.2. Phloridzin Quantification in Fresh Apple Pomace Extracts by Q-TOF-LC/MS

**Figure 4:**
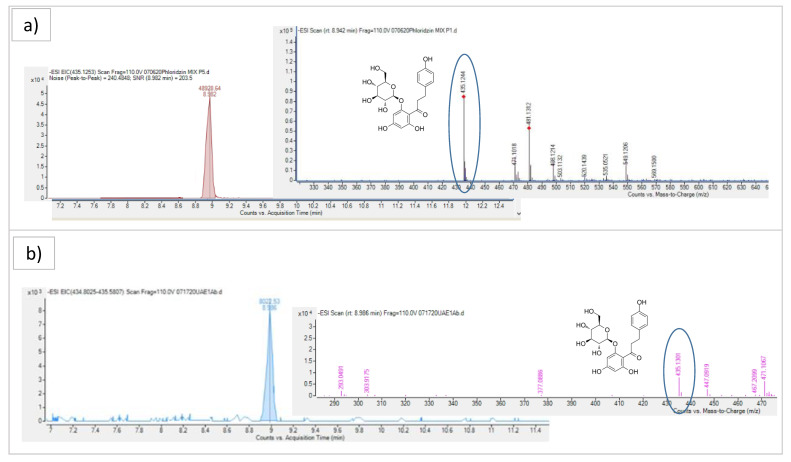
The observed chromatogram and spectrum of phloridzin in standard solution (3 µg/mL) (**a**) and UAE extract (**b**) with Q-TOF-LC/MS.

Phloridzin, a dihydrochalcone with antioxidant, anti-cardiovascular disease, and anti-diabetes effects, represents one of the most abundant and prevalent phenolic compounds in apples, so it could be used as a biomarker of authentication [23]. For this reason, the phloridzin content in the hydroalcoholic FAPE samples was evaluated, to show the varying efficiency of the extraction and to compare the different extraction methods by Q-TOF-LC/MS analysis. The observed chromatogram and spectrum of phloridzin in standard solution (3 µg/mL) (a) and UAE extract (b) are shown in Figure 4. The LD and LQ values of phloridzin were 0.04 µg/mL and 0.15 µg/mL, respectively. We report the phloridzin content, expressed as µg/g of fresh apple pomace (AP), in different FAPE samples obtained with UAE and UTE (Table 1), PEF (Table 2), and ASE (Table 3).

As regards UAE and UTE FAPE (Table 1), the content of phloridzin was very similar, despite the fact that the UAE results were slightly higher than the UTE results, ranging from 55.86 to 71.19 and from 58.39 to 64.43 of µg/g fresh apple pomace, respectively. Li et al. [24] found slightly lower content of phloridzin when extracting polyphenols by UAE treatment from different varieties of apple pulps without core and peel, ranging from 11.40 to 40.91 µg/g of fresh pulp, while Santarelli et al. [18] found lower phloridzin content when carrying out the UTE technique on freeze-dried apple pulp, ranging from 39.9 to 77.0 µg/g of dry weight.

It can be observed in Table 2 that the highest value was obtained when pre-treating the fresh apple pomace with PEF at a low intensity and for a long duration (2 kV/cm and 100 kJ/kg), using EtOH:H_2_O (70:30, *v*/*v*). Furthermore, the lowest phloridzin contents were observed in both the PEF extracts when EtOH:H_2_O (30:70, *v*/*v*) was used as the solvent mixture, followed by the EtOH:H_2_O (50:50, *v*/*v*) FAPE obtained with a moderate intensity and short duration (3 kV/cm and 17 kJ/kg).

Table 3 shows that more homogeneous results were obtained when performing the ASE technique; in particular, the values extracted at 40 °C were higher than those at 25 °C, both when 25% and 50% of flush was applied, except for the EtOH:H_2_O (50:50, *v*/*v*) samples extracted with 25% of flush, for which the opposite occurred. The temperature of 40 °C was chosen because it represents a good compromise between the increment of the extraction and the risk of degrading the phenolic compounds. Moreover, ASE performed with EtOH:H_2_O (30:70, *v*/*v*), at 40 °C and 50% of flush, was the most efficient extraction technique and gave the highest content of phloridzin (938.33 µg/g fresh AP). PEF extracts obtained with EtOH:H_2_O (70:30, *v*/*v*) and ASE extracts, especially those obtained at 40 °C, showed phloridzin contents higher than the results obtained by Fernandes et al. [25], who determined polyphenols by UHPLC-DAD after thioacidolysis (0.14 g/kg of dry apple pomace), and very similar to the ones reported by Garcia et al. [26], who found contents ranging between 0.6 and 1.5 g/kg of dry weight.

The phloridzin contents determined on Red Delicious fresh apple pomace by Q-TOF-LC/MS and TPC values were very different among them, because the method used for TPC exclusively measures the capacity of FAPE to reduce the Folin–Ciocalteu reagent and thus it is an index that measures the reducing power of the extract [21].

## 3. Materials and Methods

### 3.1. Reagents

Folin–Ciocalteu phenol reagent was purchased from Sigma-Aldrich (St. Louis, MO, USA), while sodium carbonate anhydrous was from Merck KGaA (Darmstadt, Germany). Deionized water was used throughout and obtained using a Milli-Q_PLUS_ system (Merck). Ethanol absolute, HPLC grade, Sharlau (Barcelona, Spain) and methanol for UV, IR, HPLC, ACS, PanReac Applichem (Darmstadt, Germany) were used for the extractions, while methanol OPTIMA LC/MS grade, Fisher Chemical (Madrid, Spain) was used for HPLC analysis. Catechin, chlorogenic acid, quercetin, p-coumaric acid, gallic acid, and phloridzin were purchased from Sigma-Aldrich (St. Louis, MO, USA). The individual stock solutions of phenolic compounds were prepared in MeOH:H_2_O (70:30, *v*/*v*) at 1000 µg/mL and maintained at −20 °C in the dark. Matrix-matched calibration curves at concentrations between 0.05 and 21 µg/mL were used to quantify the phenols in samples.

### 3.2. Preparation of Apple Pomace

The Red Delicious apples were acquired from a supermarket. The apples were cultivated by Ulla from the Girona region in Spain. The apple pomace was obtained, after separating seeds and petioles, with a domestic blender (Habitex Style, SC-650N blender). Aliquots were stored at −20 °C till other analyses were performed.

### 3.3. Extractions of Phenolic Compounds

#### 3.3.1. Ultrasound-Assisted Extraction (UAE)

UAE was carried out for fresh apple pomace samples according to Pollini et al. [27], with slight modifications. The following extraction conditions were used: solid/liquid ratio of 1:10 (g/mL), in an ultrasonic bath (Ultrasonic Cleaner, VWR) at 60 °C for 60 min, with EtOH:H_2_O at different ratios (50:50, 70:30, and 30:70, *v*/*v).* After centrifugation at 3000 rpm for 5 min, the obtained extract was filtered with folded qualitative filter paper (particle retention 10–20 µm, VWR), and the solvent was evaporated with gentle nitrogen flow in TurboVap Zymark at 40 °C. Then, the extracts were stored at −20 °C and resuspended in H_2_O for further analysis.

#### 3.3.2. Ultraturrax Extraction (UTE)

UTE was performed according to Rusu et al. [28]. Apple pomace samples were homogenized through Ultraturrax IKA T 18 with a 10:1 (mL/g) liquid/solid ratio, using the mixture EtOH:H_2_O at different ratios (50:50, 70:30, and 30:70, *v*/*v*) for 1 min at 9500 rpm and then 1 min at 13,500 rpm. After vortexing, the extracts were centrifuged at 3000 rpm for 15 min and filtered with folded qualitative filter paper (particle retention 10–20 µm, VWR). The solvent was evaporated with gentle nitrogen flow in TurboVap Zymark at 40 °C. Then, the extracts were stored at −20 °C and redissolved with H_2_O for further analysis.

#### 3.3.3. Accelerated Solvent Extraction (ASE)

ASE (Dionex ASE 200) was carried out by mixing the apple pomace samples with inert diatomaceous earth, in order to absorb water from the sample, at a specified sample:inert earth ratio (2:1, *w*/*w*). According to Franquin-Trinquer et al. [5], two sets of extraction, one at a temperature of 25 °C and the other at 40 °C, were performed using EtOH:H_2_O at different ratios (50:50, 70:30, and 30:70, *v*/*v*), with 3 cycles, at 1500 psi, with 25% or 50% of flush. The solvent was evaporated with gentle nitrogen flow in TurboVap Zymark at 40 °C. Then, the extracts were stored at −20 °C and redissolved with H_2_O for further analysis.

#### 3.3.4. Pulsed Electric Field Treatment (PEF)

PEF-Cellcrack III (German Institute of Food Technologies (DIL)) equipment (ELEA, Quakenbrück, Germany) was used to treat apple pomace. Samples were prepared as follows: 28.7 g of apple pomace was mixed with 200 g of tap water, and the gap between the electrodes was set at 10 cm. Two experiments were performed, in line with Lohani et al. [21], the first one with a specific energy input of 100 kJ/kg and the second one of 17 kJ/kg; the number of pulses was 115 and 9 pulses, depending on the voltage applied (field strength 2 or 3 kV/cm, respectively). These conditions were derived from the fact that a minimum of 1 kV/cm field strength is required to produce changes in cells and 3–4 kV/cm for the electroporation [29]. For this reason, only two experiments were carried out in order to compare a treatment of moderate intensity and short duration (3 kV/cm and 9 pulses with an energy per pulse of 450 J) with another one of low intensity and long duration (2 kV/cm and 100 pulses with an energy per pulse of 200 J). Before and after treatment, the temperature and conductivity were measured in the sample with a portable conductivity meter ProfiLine Cond 3310 (WTW, Xylem Analytics Weilheim in Oberbayern, Germany). The PEF-treated samples were freeze-dried and then phenolics were extracted using a liquid/solid ratio of 10:1 (mL/g), EtOH:H_2_O at different ratios (50:50, 70:30, and 30:70, *v*/*v*), low shaking for 1 h, followed by centrifugation at 4500 rpm for 10 min and filtration with folded qualitative filter paper (particle retention 10–20 µm, VWR). The solvent was evaporated with gentle nitrogen flow in TurboVap Zymark at 40 °C. Then, the extracts were stored at −20 °C and redissolved with H_2_O for further analysis.

### 3.4. Determination of the Total Phenolic Content (TPC)

The spectrophotometric determination of TPC of apple extracts was carried out according to Montesano et al. [30], using 20% Na_2_CO_3_ water solution and Folin–Ciocalteu reagent 2 N. After 30 min of reaction in the dark, the absorbance at λmax = 750.0 nm was read using a UV–Vis Cecil Super Aquarius CE 9500 spectrophotometer. Different gallic acid solutions were used to create a calibration curve and the results were expressed as microgram of gallic acid equivalent per gram of fresh apple pomace (µg GAE/g of fresh apple pomace AP).

### 3.5. Q-TOF-LC/MS Analysis of Phenolic Compounds

The chromatographic ultra-high-performance Accurate-Mass Q-TOF-LC/MS analysis was performed using an Agilent Technologies (Santa Clara, CA, USA) 1200 Infinity Series LC coupled with an Agilent Technologies 6540 UHD Accurate-Mass Q-TOF-LC/MS. This device was equipped with an electrospray ionization Agilent Technologies Dual Jet Stream ion source (Dual AJS ESI). Chromatographic separation was carried out with an Agilent Infinity lab Poroshell 120 EC-C18 (3 × 100 mm, 2.7 µm) column. The injection volume was 20 µL. The mobile phase consisted of 0.1% formic acid in water milli-Q (solvent A) and 0.1% formic acid in acetonitrile (solvent B), at a flow rate of 0.5 mL/min, with the following gradient: 0–10 min, 5% B; 10–13 min, 95% B; 13–15 min, 95% B. The Q-TOF-MS conditions were the following: drying gas flow (N_2_), 12.0 L/min; nebulizer pressure, 45 psi; gas drying temperature, 370 °C; capillary voltage, 3500 V; fragmentor voltage, 110 V; skimmer voltage 65 V, and octopole RF peak, 750 V. Dual AJS ESI interface was used in negative ionization mode and negative ions were acquired in the range of 100–1100 *m*/*z* for MS scans, and 50–600 *m*/*z* for auto MS/MS scans, at a scan rate of 5 scans/s for MS and 3 scans/s for MS/MS, respectively. Automatic acquisition mode MS/MS was carried out using the following collision energy values: *m*/*z* 20 eV; *m*/*z* 30 eV, and 40 eV. Internal mass correction was enabled, by using two reference masses at 121.0509 and 922.0098 *m*/*z*. Instrument control and data acquisition were performed using Agilent MassHunter Workstation software B.08.00. All the MS and MS/MS data of the validation standards were integrated by MassHunter Quantitative Analysis B.10.0 (Agilent Technologies).

### 3.6. Standard Preparation, Calibration Curves, Limits of Detection, and Quantification

A methanol stock solution containing all six reference standards (gallic acid, catechin, chlorogenic acid, quercetin, p-coumaric acid, and phloridzin) was prepared by dissolving the reference standards in methanol to a final concentration of 1 mg/mL each; then, we diluted the mixture stock solution to an appropriate concentration to establish calibration curves. The matrix-matched calibration solutions (3.12, 6.25, 12.5, 25, and 50 μg/mL) were prepared by combining the working solution with blank matrix solution. All of the solutions were stored at −20 °C before use. All calibration curves were constructed from peak areas of reference standards versus their concentrations. The lowest concentration of working solution was diluted with methanol to yield a series of appropriate concentrations. According to the International Conference on Harmonization (ICH, 2005), the limit of detection (LOD) and limit of quantification (LOQ) were calculated based on matrix-matched calibration as the concentration for which S/N were 3 and 10, respectively. Quantifier ions were [M−H]^−^ for negative mode with a mass accuracy window of 5 ppm. Figure S1 shows the chromatographic separation of each analyzed phenolic compound in a standard solution of 3 µg/mL.

### 3.7. Statistical Analysis

The results were reported as mean values, based on two replicates, ±standard deviation. Microsoft Excel 2016 (Microsoft Corporation, Redmond, WA, USA) was used for data analysis.

## 4. Conclusions

Based on the obtained results, it is possible to determine that UAE is the best extraction technique to evaluate the TPC using EtOH:H_2_O (50:50, *v*/*v*), while ASE at 40 °C was the most efficient extraction method to recover phloridzin. Therefore, ASE could be considered a powerful tool to isolate and Q-TOF-LC/MS to identify and quantify phloridzin as an important biomarker for apple pomace in food industry and food quality. In the context of the circular economy, it is interesting to investigate the most efficient extraction techniques to isolate phenolic compounds from food waste and give them new life in the development of a value-added product. This work is an important starting point to valorize apple pomace, a very cheap and common by-product, that is obtained in tons during juice production. Furthermore, apple pomace could be considered a valuable type of waste, as a source of numerous bioactive compounds, such as polyphenols, for the development of functional foods and nutraceuticals.

## Figures and Tables

**Table 1:** Phloridzin content (µg/g fresh AP, mean ± SD n = 2) obtained with UAE and UTE.

Conditions	UAE	UTE
EtOH:H_2_O (*v*/*v*)	60 °C, 60 min	1 min at 9500 rpm, 1 min at 13,500 rpm
50:50	71.19 ± 26.34	64.43 ± 23.84
70:30	63.63 ± 23.76	62.23 ± 23.21
30:70	55.86 ± 21.62	58.39 ± 21.90

**Table 2:** Phloridzin content (µg/g fresh AP, mean ± SD n = 2) obtained with PEF.

Conditions	PEF	
EtOH:H_2_O (*v*/*v*)	20.0 kV–100 kJ/kg	30.0 kV–17 kJ/kg
50:50	65.21 ± 2.11	17.56 ± 0.63
70:30	753.84 ± 26.38	208.53 ± 7.30
30:70	9.29 ± 0.34	11.49 ± 0.43

**Table 3:** Phloridzin content (µg/g fresh AP, mean ± SD n = 2) obtained with ASE.

Conditions	25% FLUSH		50% FLUSH	
EtOH:H_2_O (*v*/*v*)	25 °C	40 °C	25 °C	40 °C
50:50	654.10 ± 7.85	401.98 ± 16.14	533.37 ± 48.00	782.84 ± 13.31
70:30	393.86 ± 4.21	513.42 ± 20.54	314.23 ± 29.16	388.06 ± 6.21
30:70	271.07 ± 2.98	653.63 ± 26.15	140.20 ± 12.44	938.33 ± 15.60

## Supplementary Materials

The following are available online, Figure S1: Chromatographic separation of each analyzed phenolic compound in a standard solution of 3 µg/mL by Q-TOF-LC/MS.
