# Supplementary material for: Extraction of Phenolic Compounds from Fresh Apple Pomace by Different Non-Conventional Techniques
Source: Molecules. 2021 Jul 14;26(14):4272. doi: 10.3390/molecules26144272 (PMC8307736; doi:10.3390/molecules26144272)
Supplement: Supplementary file 1 [file molecules-26-04272-s001.zip › molecules-1285360-supplementary.pdf]

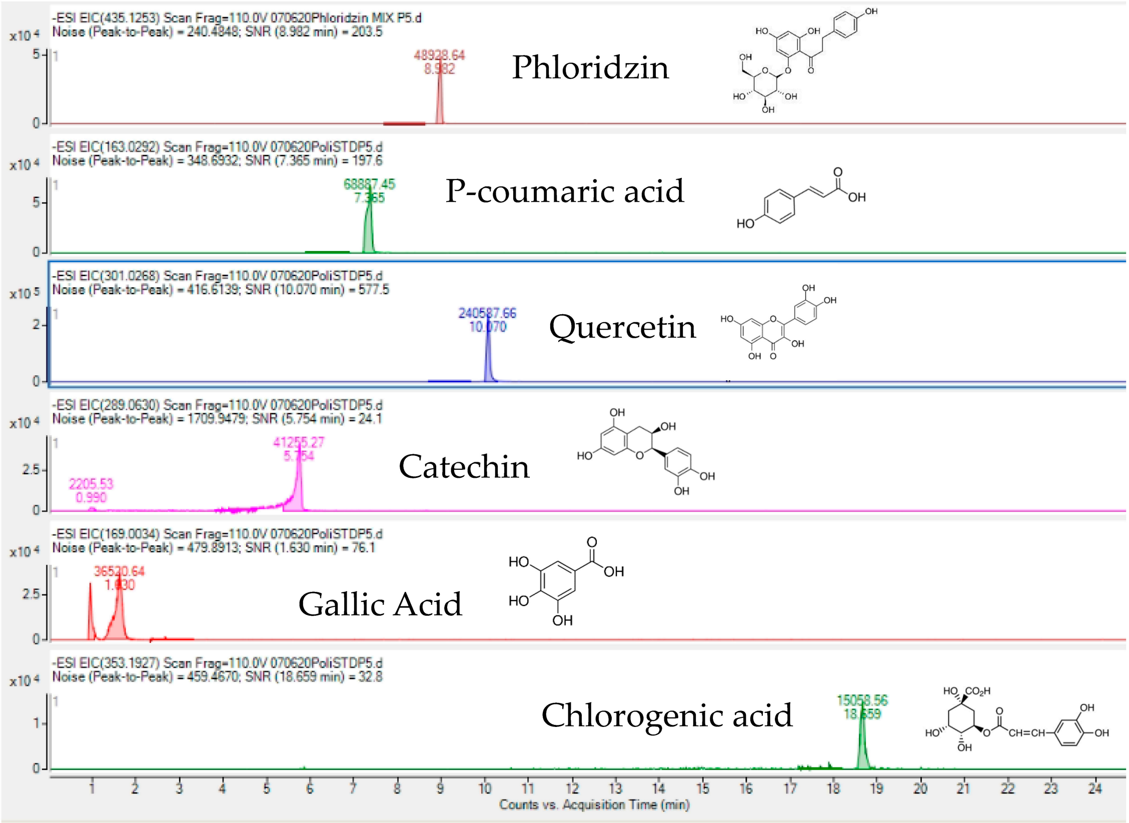

Figure S1. Chromatographic separation of each analyzed phenolic compound in a standard solution of 3 µg/mL by Q-TOF-LC/MS.
